# Association of the hypoxia-inducible factor-1α (HIF-1α) gene polymorphisms with prognosis in ovarian clear cell carcinoma

**DOI:** 10.1186/s13048-019-0481-9

**Published:** 2019-01-24

**Authors:** Hiroyuki Suzuki, Mitsutake Yano, Mariko Miyazawa, Masaki Miyazawa, Naoki Ogane, Kosei Hasegawa, Hitoshi Tsuda, Masayuki Yoshida, Ryugo Okagaki, Osamu Ishihara, Masanori Yasuda

**Affiliations:** 1 Department of Pathology, Saitama Medical University International Medical Center, Saitama, Japan; 2 Department of Obstetrics and Gynecology, Saitama Medical University Hospital, Saitama, Japan; 3 Department of Obstetrics and Gynecology, Oita University Faculty of Medicine, Oita, Japan; 4 Department of Obstetrics and Gynecology, Tokai University School of Medicine, Kanagawa, Japan; 5 Division of Pathology, Ashigarakami Hospital, Kanagawa, Japan; 6 Department of Gynecologic Oncology, Saitama Medical University International Medical Center, Saitama, Japan; 7 Department of Basic Pathology, National Defense Medical College, Saitama, Japan; 8 Department of Pathology and Clinical Laboratories, National Cancer Center Hospital, Tokyo, Japan

**Keywords:** Ovarian clear cell carcinoma, Single-nucleotide polymorphisms (SNPs), Hypoxia-inducible factor-1α (HIF-1α), Prognosis

## Abstract

**Background:**

Ovarian clear cell carcinoma (OCCC) is the second most common ovarian cancer after serous carcinoma in Japan. OCCC has a more unfavorable clinical outcome due to a poor response to platinum-based chemotherapy, compared with serous carcinoma. Hypoxia inducible factor-1α (HIF-1α) is a key regulator of cellular response to hypoxia and plays an important role in tumor growth, and *HIF-1α* gene single-nucleotide polymorphisms (SNPs) adversely affect the outcome in some cancers. Herein, we investigated the association of the *HIF-1α* gene SPNs with clinical outcome in OCCCs. Eighty-nine patients with OCCC were recruited in whom pathological diagnosis was confirmed with surgically resected specimen.

**Results:**

The SNPs of C1772T and G1790A in the *HIF-1α* gene occurred in 23.6 and 3.3% of the patients, respectively. In the univariate analysis, overall survival was associated with stage and surgical residual tumor but not with the SNPs C1772T, G1790A, C1772T and/or G1790A. In the multivariate survival analysis, a significant association was observed between outcome and FIGO stage and/or surgical residual tumor; however, no association was obtained between *HIF-1α* gene SNPs and these factors.

**Conclusion:**

In conclusion, unlike the other cancers in which *HIF-1α* gene SNPs were demonstrated to be associated with the outcome, OCCC prognosis may not be affected by *HIF-1α* gene SNPs. Further studies need to be performed to clarify the association of HIF-1α expression with the unfavorable prognosis in OCCCs, in terms of transcriptional/translational activity, nuclear translocation of the protein, and protein degradation.

## Background

Ovarian cancer is the leading cause of death among gynecological malignancies, as well as is the fourth most common malignancy in women in developed countries, following breast, lung, and colorectal cancer [1, 2]. Each of the ovarian cancers, represented by serous carcinoma, endometrioid carcinoma, clear cell carcinoma, and mucinous carcinoma, are known to have specific clinicopathological features and molecular or genetic characteristics. In Japan, ovarian clear cell carcinoma (OCCC) is the second most common ovarian cancer, following serous carcinoma [3, 4]. OCCC arises from endometriosis in 50–70% of the cases [5, 6] and has a more unfavorable prognosis due to a poor response to platinum-based chemotherapy, compared with serous carcinoma [3, 4].

HIF-1α is a key regulator of cellular response to hypoxia and plays an important role in tumor growth by trans-activating various genes that are related to regulation of angiogenesis, energy metabolism, survival, resistance to anti-tumor therapy, and cell survival, apoptosis, and proliferation [7–9]. In our previous studies of OCCC and other ovarian epithelial cancers, we found an increased nuclear expression of HIF-1α in OCCC and have identified the HIF-1α regulating factors [10, 11]. Genetic polymorphisms are responsible for inter-individual variation and diversity, and have been recently considered as the main genetic elements involved in the development and progression of cancer [12]. *HIF-1α* gene SNPs are more frequent in several cancers than in healthy groups [13–29]. Furthermore, they are associated with a poor prognosis in some cancers, including non-small cell lung cancer [13, 14], breast cancer [15, 16], head and neck squamous cell carcinoma [17], prostate cancer [18], bladder cancer [19], and glioma [20]. A total of 35 SNPs have been located within the *HIF-1α* gene. Three of the 35 SNPs were located in coding regions, one in exon 2, and the others in exon 12 [30]. The two SNPs located within exon 12 (codon 582 and 588) were associated with transcriptional activity [9, 30]. The C to T transition at nucleotide 1772 leads to an amino acid change of proline to serine at codon 582 (C1772T/P582S/rs11549465), and the G to A nucleotide substitution at point 1790 gives rise to an alanine/threonine variation at codon 588 (G1790A/A588T/rs11549467).

This study was conducted to investigate the impact and susceptibility of HIF-1α gene SNPs (C1772T and G1790A) on the prognosis of OCCCs because there have been no reports to analyze the association of the SNPs with outcome. In particular, the two SNPs associated with transcriptional activity were the focus of the study because they were associated with transcriptional activity.

## Results

**Fig. 1:**
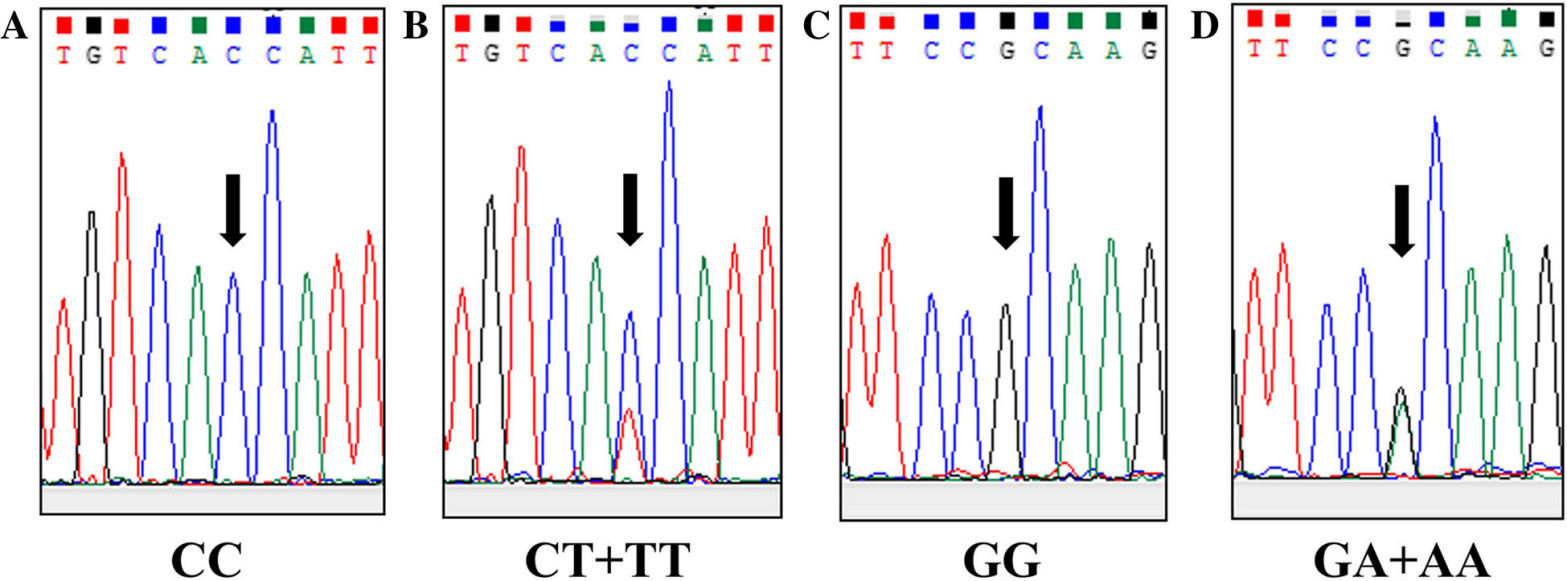
Polymorphisms in the *HIF-1α* gene: Chromatograms of DNA sequence analysis of *HIF-1α* showing the allelic variations at position 1772 and 1790. (**a**) Homozygous wild-type SNP C1772T (CC), (**b**) heterozygous/homozygous SNP C1772T (CT + TT), (**c**) homozygous wild-type SNP G1790A (GG), and (**d**) heterozygous/homozygous SNP G1790A (GA + AA)

The genotypes of the homozygous wild-type *HIF-1α* gene SNPs C1772T (CC) and G1790A (GG) as well as heterozygous/homozygous SNPs C1772T (CT + TT) and G1790A (GA + AA) were identified (Fig. 1). Among the 89 OCCC patients, 23.6 and 3.3% showed the presence of C1772T and G1790A SNPs in the *HIF-1α* gene, respectively. Results were compared with those for the Japanese healthy population group; prevalence of C1772T and G1790A SNPs was 9.1–11.0% and 8.2–8.7%, respectively [19, 31–33]. All clinicopathological results (age, FIGO stage, surgical residual tumor, recurrence, and death) failed to show a significant relationship with the SNPs (Table 1).

**Table 1:** Associations of the HIF-1α polymorphisms with clinicopathological parameters of OCCC

		C1772T			G1790A			C1772T and/or G1790A		
Variable	N (%)	CC	CT + TT	*p* value	GG	GA + AA	*p* value	CC and GG	CC + TT and/or GA + AA	*p* value
Age										
≥ 54	43 (48)	33	10	0.571	41	2	0.474	31	12	0.518
< 54	46 (52)	35	11		45	1		34	12	
FIGO stage										
I and II	70 (79)	55	15	0.262	68	2	0.518	53	17	0.209
III and IV	19 (21)	13	6		18	1		12	7	
Residual tumor										
Yes	11 (12)	9	2	0.493	10	1	0.330	8	3	0.616
No	71 (88)	59	19		76	2		57	21	
Recurrence										
Yes	23 (26)	19	4	0.306	22	1	0.597	18	5	0.358
No	66 (74)	49	17		64	2		47	19	
Death										
Yes	15 (17)	12	3	0.506	14	1	0.429	11	4	0.625
No	74 (83)	56	18		72	2		54	20	

*CC* 1772CC genotype, *CT* 1772CT genotype, *TT* 1772TT genotype, *GG* 1790GG genotype, *GA* 1790GA genotype, *AA* 1790AA genotype, *FIGO* the International Federation of Obstetrics and Gynecology

**Fig. 2:**
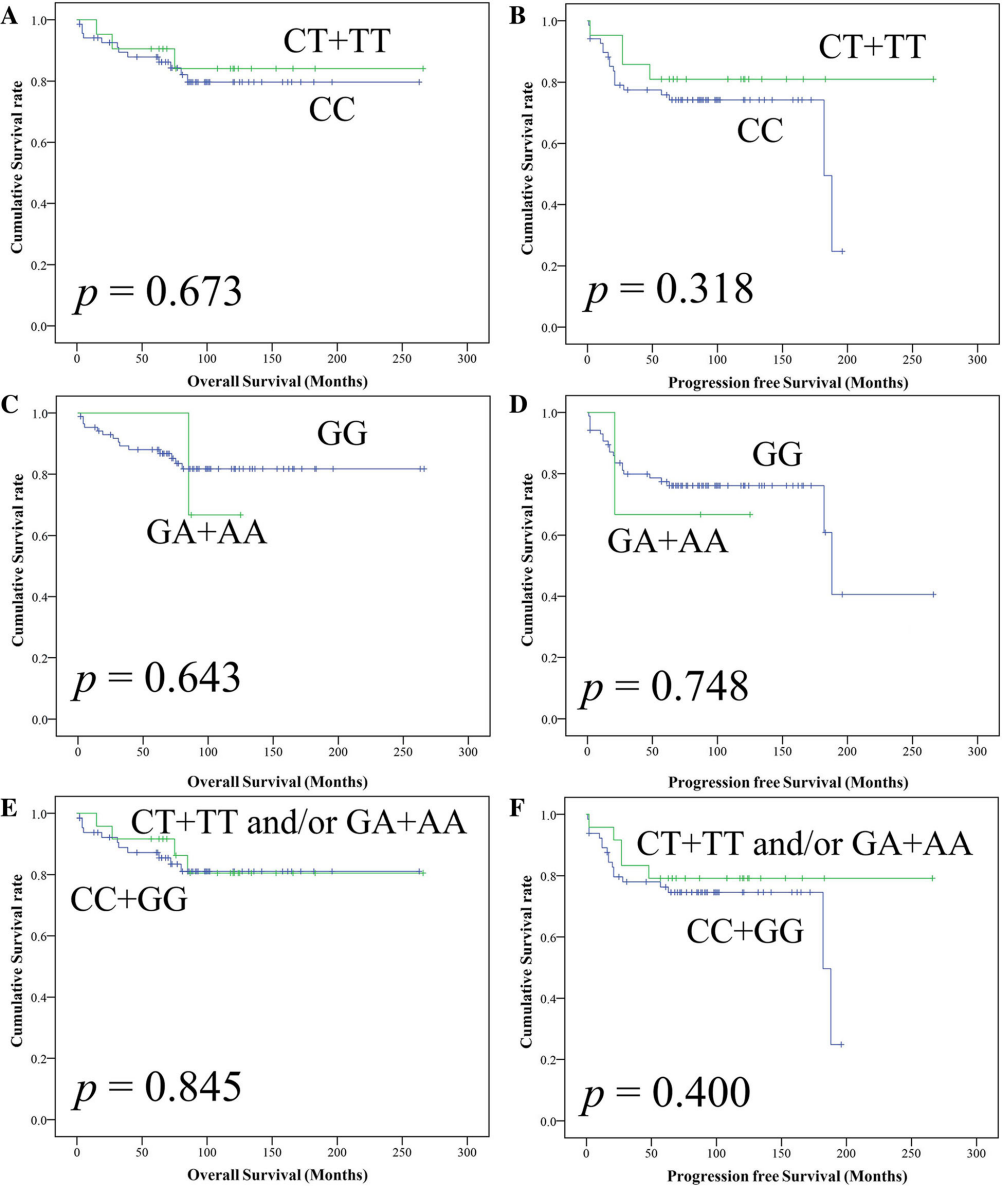
Kaplan-Meier survival analysis: Overall survival of OCCC patients according to the (**a**) C1772T, (**b**) G1790A, and (**c**) C1772T and/or G1790A. Progression free survival of OCCC patients according to the (**d**) C1772T, (**e**) G1790A, and (**f**) C1772T and/or G1790A. *p* values, log rank test

In Kaplan-Meier survival curves, C1772T SNPs (CT + TT genotype) had no significant adverse effect on OS (*p* = 0.673, Fig. 2a) and PFS (*p* = 0.318, Fig. 2b). G1790A SNPs (GA + AA genotype) also had no significant adverse effect on OS (*p* = 0.643, Fig. 2c) and PFS (*p* = 0.748, Fig. 2d). Additionally, C1772T and/or G1790A SNPs (CT + TT and/or GA + AA) had no significant adverse effect on OS (*p* = 0.845, Fig. 2e) and PFS (*p* = 0.400, Fig. 2f). However, FIGO stage and surgical residual tumor had a significant adverse effect on OS (*p* = < 0.001; *p* = < 0.001, respectively) and PFS (*p* = < 0.001; *p* = < 0.001, respectively).

In the univariate analysis using the Cox proportional hazard model, OS was associated with FIGO stage (hazard ratio (HR) = 15.62; 95% confidence interval (CI) = 4.949 to 49.31; *p* = < 0.001) and surgical residual tumor (HR = 16.13; 95% CI = 5.780 to 45.00; *p* = < 0.001), but not with C1772T (HR = 0.762; 95% CI, 0.215 to 2.701; *p* = 0.674), G1790A (HR = 1.609; 95% CI, 0.211 to 12.28; *p* = 0.647), C1772T and/or G1790A (HR = 0.892; 95% CI, 0.284 to 2.803; *p* = 0.845), and age (HR = 1.463; 95% CI, 0.529 to 4.049; *p* = 0.463) (Table 2). In the multivariate survival analysis, FIGO stage (HR = 7.527; 95% CI, 1.808 to 31.33; *p* = 0.006) and surgical residual tumor (HR = 4.030; 95% CI, 1.127 to 14.41; *p* = 0.032) were found to be the independent prognostic factors (Table 2).

**Table 2:** Univariable and multivariable analysis using the Cox proportional hazards model of overall survival for OCCCs (*n* = 89/15 events)

	Univariate analysis			Multivariate analysis		
Variable	HR	95% CI	*p* value	HR	95% CI	*p* value
C1772T	0.762	0.215–2.701	0.674			
G1790A	1.609	0.211–12.28	0.647			
C1772T and/or G1790A	0.892	0.284–2.803	0.845			
Age (> 54 vs ≤54)	1.463	0.529–4.049	0.463			
FIGO stage (III + IV vs I + II)	15.62	4.949–49.31	< 0.001	7.527	1.808–31.33	0.006
Residual tumor	16.13	5.780–45.00	< 0.001	4.030	1.127–14.41	0.032

*HR* Hazard ratio, *CI* confidence interval, *FIGO* the International Federation of Obstetrics and Gynecology

## Discussion

HIF-1α expression represents an important biomarker in the evaluation of ovarian carcinoma prognosis [34]. In our study, OCCCs are characterized by a nuclear expression of HIF-1α compared to other histological types. It is believed that HIF-1α is one of the key factors closely associated with chemo-resistance or unfavorable OCCC prognosis [10, 11]. Overexpression of HIF-1α may be attributed to transcriptional and/or translational activity, nuclear transition of the protein, and its degradation.

This study was conducted to assess whether there is an association of the *HIF-1α* gene SNPs with the prognosis and clinicopathological characteristics of OCCCs. A significant association was observed between prognosis and clinicopathological factors such as FIGO stage and surgical residual tumor. However, any variations of the SNPs were proven not to be associated with the prognosis. The previous studies of variable cancers with a focus on the relationship between *HIF-1α* gene SNPs and patient prognosis are summarized in Table 3 [13–29]. OCCC patients had more frequent C1772T SNPs than the healthy Japanese population [19, 31–33] and many other carcinomas. OCCC prognosis as well as colorectal cancer [21, 22], thymic malignancy [27], and cervical cancer [28, 29] prognoses had no association with C1772T and G1790A SNPs. However, the T allele of C1772T and A allele of G1790A are a poor or good prognostic factor in several cancers [17, 23]. The effects of *HIF-1α* SNPs on the prognosis with cancers are not uniform.

**Table 3:** *HIF-1α* polymorphisms in various cancers

Type of cancer (Reference No.)	Case	Frequency (%)		Prognosis	
		C1772T	G1790A	C1772T	G1790A
OCCC (present study)	89	23.6	3.3	No association	No association
Colorectal cancer (21)	336	20.6	2.7	No association	No association
Colorectal cancer (22)	445	7.9	7.0	No association	No association
NSCLC (13)	741	73.5	72.5	CC has longer survival than CT and TT	No association
NSCLC (14)	285	46.3	47.4	TT has shorter survival than CC and CT	AA has shorter survival than GG and GA
Breast cancer (15)	90	10.0	3.3	C1772T polymorphism is associated with HIF-1α overexpression, found in patients with lymph node metastasis	No association
Breast cancer (16)	410	28.2	19.1	T allele increases risk for lymph nodes metastasis	No association
Prostate cancer (18)	754	21.9	NA	T allele increases risk for metastasis and resistance to ADT	NA
RCC (23)	160	90.0	55.5	TT is earlier stage than the CC and CT	No association
RCC (24)	620	7.7	7.3	No association	No association
HNSCC (17)	52	50.0	71.2	T allele is more frequently found in patients with metastasis	GA and GG have shorter survival than AA
OSCC (25)	305	7.5	7.9	No association	No association
OSCC (26)	74	18.6	37.5	No association	A allele has shorter survival
Thymic malignancy (27)	57	14.9	0	No association	NA
Bladder cancer (19)	219	10.0	6.8	C1772T and/or G1790A polymorphic variants have shorter survival	
Cervical cancer (28)	162	14.2	6.8	No association	No association
Cervical cancer (29)	199	11.1	6.0	No association	No association
Glioma (20)	387	70.5	75.2	CC has longer survival than CT and TT	No association

*OCCC* ovarian clear cell carcinoma, *NSCLC* non-small cell lung cancer, *CC* 1772CC genotype, *CT* 1772CT genotype, *TT* 1772TT genotype, *GG* 1790GG genotype, *GA* 1790GA genotype, *AA* 1790AA genotype, *NA* not available, *RCC* renal cell carcinoma, *HNSCC* head and neck squamous cell carcinoma, *OSCC* oral squamous cell carcinoma

The C1772T SNP has been reported to increase HIF-1α protein expression in some cancers [13, 15]. Twenty specimens, which were randomly selected out of the 89 OCCCs examined in this study, were subjected to immunohistochemical staining for HIF-1α. The results failed to show the associated between HIF-1α staining and presence of SNPs (data not shown). In our previous studies, OCCCs showed the highest frequency of HIF-1α, histone deacetylase (HDAC) 6, and HDAC7 compared to other ovarian epithelial cancer [10, 11, 35]. HDAC6 and HDAC7 induced not only HIF-1α transcriptional activity, but also stabilized HIF-1α protein via interaction with von Hippel Lindau and ubiquitin-independent proteasomal degradation of HIF-1α [36–38]. In OCCCs, post-translational modification may be more important for the HIF-1α expressions than upregulated transcription activity by *HIF-1α* gene SNPs.

Our study has several limitations. The sample size used in this study was small and the survival analysis was only performed with a few events. However, when considering the low incidence of OCCC, the present study included a relatively large number of patients. Secondly, normal controls were not recruited in the present study; instead, we compared the frequencies of *HIF-1α* SNPs using the normal Japanese population reported in the past studies [19, 31–33].

## Conclusion

In conclusion, *HIF-1α* gene SNPs were demonstrated to be less significant as a prognostic marker in OCCCs. The precise mechanism of the association between the SNPs and overexpression of protein level remains to be clarified.

## Methods

### Patient data and clinicopathological features (Table 4)

Patients’ electronic medical charts from the Saitama Medical University Hospital and Saitama Medical University International Medical Center during the period of 1994 to 2012 were reviewed under approval of the institutional review board (IRB) following the ethical standards of the responsible committee on human experimentation and with the revised Helsinki Declaration in 1983. A total of 89 patients with OCCC without preoperative chemotherapy, whose tumors were surgically resected and pathologically confirmed, were recruited for this study. Clinicopathological characteristics of these cases, such as age, the International Federation of Obstetrics and Gynecology (FIGO) stage, treatment methods, recurrence, death, progression free survival (PFS), and overall survival (OS) were reviewed.

**Table 4:** Clinicopathological characteristics of patients (*n* = 89)

Variable	N (%)
Age	
Median (range)	54.4 (34–78)
> 54	43 (48)
≤ 54	46 (52)
FIGO stage	
I	65 (73)
II	5 (6)
III	17 (19)
IV	2 (2)
Treatment	
OP only	18 (20)
OP+AC	71 (80)
Recurrence	
Yes	23 (26)
No	66 (74)
Death	
Yes	15 (17)
No	74 (83)

*FIGO* the International Federation of Obstetrics and Gynecology, *OP* operation (at least primary tumor resection), *AC* adjuvant chemotherapy

### Genotyping of *HIF-1α* single-nucleotide polymorphism

Samples were recruited from the formalin-fixed, paraffin embedded surgical specimens of OCCCs. DNA was extracted using the Gentra Puregene Tissue Kit (Qiagen, Germantown, MD, USA) according to the manufacturer’s instructions. Polymerase chain-reaction (PCR) was performed using the following specific primers designed for exon 12: 5′-GCTCCCTATATCCCAATGGA-3′ (forward) and 5′-CAGTGGTGGCAGTGGTAGTG-3′ (reverse). The PCR conditions applied were: 1 cycle of 95 °C for 2 min, followed by 40 cycles of 94 °C for 30 s, 60 °C for 30 s, and 1 min at 72 °C with final extension at 72 °C for 10 min. For each assay, a negative control (without DNA template) was added to monitor PCR contaminations. After confirming the integrity of the amplicons, all PCR products were further purified using ExoSAP-IT PCR Product Clean-up (Affymetrix, Santa Clara, CA, USA) for commercial sequencing. The sequencing primer was the same as the forward primer used for the PCR reaction. Biosystems 3130 Genetic Analyzer (Applied Biosystems, Foster City, CA, USA) was used for reading sequences on the chromatograms.

### Statistical analysis

Genetic polymorphisms and clinic pathological parameters were assessed using the Pearson chi-square test or the Fisher exact test. Univariable survival analysis was performed by the generation of Kaplan-Meier curves, and differences between the groups were assessed using the log rank statistic. Univariable and multivariable survival analyses were performed using the Cox proportional hazards model. SPSS v24.0 (SPSS Inc., Chicago, IL, USA) was applied for these all analyses. *p* values < 0.05 were considered significant.

## Abbreviations

CI: Confidence interval
FIGO: The International Federation of Obstetrics and Gynecology
HDAC: Histone deacetylase
HIF-1α: Hypoxia inducible factor-1α
HR: Hazard ratio
IRB: Institutional review board
OCCC: Ovarian clear cell carcinoma
OS: Overall survival
PCR: Polymerase chain-reaction
PFS: Progression free survival
SNP: Single-nucleotide polymorphism
